# Acute Mania and Catatonia in a Teenager Successfully Treated with Electroconvulsive Therapy and Diagnosed with Turner Syndrome and Bipolar Disorder

**DOI:** 10.1155/2021/3371591

**Published:** 2021-12-17

**Authors:** Maria Ygland Rödström, Björn Axel Johansson, Beata Bäckström, Pouya Movahed, Carl-Magnus Forslund, Olof Rask

**Affiliations:** ^1^Division of Emergency Medicine, Karolinska University Hospital, Stockholm, Sweden; ^2^Region Skåne, Psychiatry, Habilitation & Aid, Child and Adolescent Psychiatry, Regional Inpatient Care, Emergency Unit, Malmö, Sweden; ^3^Department of Clinical Sciences Lund, Division of Child & Adolescent Psychiatry, Lund University, Lund, Sweden; ^4^Region Skåne, Psychiatry, Habilitation & Aid, Child and Adolescent Psychiatry, Unit for Pediatric Bipolar & Psychotic Disorders, Lund, Sweden; ^5^Department of Clinical Sciences, Faculty of Medicine, Lund University, Lund, Sweden

## Abstract

**Background:**

Turner syndrome (TS) is an X-linked chromosomal abnormality with a global prevalence of 1/2000 live-born girls. The physiological symptoms of TS have been thoroughly characterized, but only a few studies have described associated psychiatric symptoms. We report a case of an adolescent girl who presented with acute mania with psychotic features and was successfully treated with electroconvulsive therapy (ECT). She was subsequently diagnosed with bipolar syndrome and TS. *Case Presentation*. A 17-year-old girl presented to us with manic symptoms, including disorganized speech, auditory hallucinations, and affect lability. Initially, she was treated with antipsychotics and benzodiazepines, whereby the positive affective symptoms declined. However, the psychotic symptoms progressed, and she developed a catatonic state. ECT was started 6 days after admission, with improvement after two treatments. When ECT was tapered after seven sessions, she relapsed, and the treatment was extended to twelve sessions, with successful outcome. Following discharge, she was diagnosed with TS with partial loss on one of the X-chromosomes (46X, del (X)(p21)), which might have contributed to the development of her sudden acute manic episode.

**Conclusions:**

This case demonstrates for the first time that ECT may be a safe and efficient treatment strategy for acute mania in adolescents with concomitant TS and that severely affected adolescents may require a prolonged series with gradual tapering of ECT. The present case also demonstrates a possible association between TS and bipolar syndrome and that the clinical presentation of a manic episode in a patient with this comorbidity could be more complex and the treatment response slower.

## 1. Introduction

TS is caused by a chromosomal abnormality where a partial or a complete copy of the X-chromosome is missing in some or all cells [1]. TS has a global prevalence of 1 per 2000 newborn girls [2–4]. The syndrome, first described by Henry Turner in 1938, is typically characterized by a short or webbed neck, low-set ears, obesity, and a short stature. TS is heterogeneously associated with a range of disorders, including infertility, heart conditions, skeletal abnormalities, Hashimoto's thyroiditis, and diabetes mellitus [1]. Regarding the wide symptom range, the presentation of TS varies among the affected individuals. A majority of persons with TS have preserved general intelligence and verbal skills but often have deficits in visuospatial function and arithmetic and executive functions and may have difficulties with scholastics and social interactions [5].

Bipolar syndromes involve extreme fluctuation in mood, energy, and ability to function according to the Diagnostic and Statistical Manual of Mental Disorders 5 (DSM) [6]. In bipolar I disorder, the criteria for a manic episode must be met, which can exist with or without psychosis [6].

TS is associated with an increased risk for various neuropsychiatric disorders, including attention deficit hyperactivity disorder (ADHD), autism spectrum disorders, depression, schizophrenia, and high anxiety levels [1, 5, 7, 8]. A connection between X-chromosomal gene expression and bipolar syndrome has been investigated, and some studies have found an association to the X-chromosome, but still, no clear causal association has been found [9]. Research conducted on TS with bipolar disorder is scarce, and the few published results of small studies and case reports have been heterogeneous; hence, the prevalence of bipolar disorder in TS patients is still unknown [10–12]. In a review by Catinari et al., it was proposed that a subset of patients with TS could have a genetic vulnerability to certain psychiatric conditions such as psychosis, often triggered by stressful situations [10]. Still, little is known about the clinical presentation or response to treatment for this group of patients or the association between TS and acute manic episodes.

Electroconvulsive therapy (ECT) is considered a safe and effective treatment strategy for adults with severe depression, catatonia, and manic episodes [13, 14]. However, ECT is more seldom used for children or adolescents, although similarly considered safe and efficient [15]. To our knowledge, there is no published case report on ECT in TS patients.

In this case report, we describe a 17-year-old girl who presented with persistent symptoms of acute mania with psychotic features and catatonia. She showed no improvement despite treatment with antipsychotics and benzodiazepines, after which ECT was initiated. At follow-up, our patient was diagnosed with bipolar disorder and TS with a partial loss on one of the X-chromosomes (46X, del (X)(p21)). This case demonstrates important diagnostic and treatment challenges with concomitant acute manic episodes and X-linked diseases such as TS, which is important for pediatric psychiatrists and neurologists to consider.

## 2. Case Presentation

### 2.1. Previous History

A 17-year-old girl from Sweden was presented to us at the Child and Adolescent Psychiatry Emergency Unit during spring 2018 with acute manic and psychotic symptoms. She was previously physically healthy except for irregular menstruation and menorrhagia with one occasion of anemia, for which she was treated. She had no history of substance use disorder. In the family history, it was noted that her maternal grandmother had schizophrenia and her grandmothers' mother had committed suicide. She had always had a limited number of friends, and according to her parents, she had been academically motivated up to the 9^th^ grade at 15 years of age and, like her parents, had a musical talent. At 16 years of age, after a year at a competitive music high school, she had to restart with a less ambitious program due to anxiety and a period of obsessive-compulsive disorder. During autumn before admission, our patient experienced fatigue, apathy, sleeping problems, and depressed mood. She was diagnosed with mild depression in December 2017 and received psychosocial support in an outpatient unit. She was prescribed melatonin, improved, and returned to school in the early spring of 2018 with reduced study-pace but still trailed academically.

In February, there was a sudden change in mood and energy from the depressive state to a state with hypomanic symptoms. The parents noticed that, over a period of weeks, their daughter had more energy and elevated mood and became more talkative and social. The parents also described that she developed greater self-esteem, became overly ambitious, and made unrealistic plans. A few days before admission, she experienced arousal, had an increased speech flow, unmotivated laughs, neglected hygiene routines, and ate and slept less than usual. Following a few days with very little sleep and anxiety, she woke up agitated and with delusions of having her brothers inside her and controlling her mind. She tried to jump from the apartment balcony but was stopped by her parents, who called an ambulance, which in turn required police assistance to take her to the hospital in a very agitated mood.

### 2.2. Inpatient Care and Course of Acute Illness

**Figure 1:**
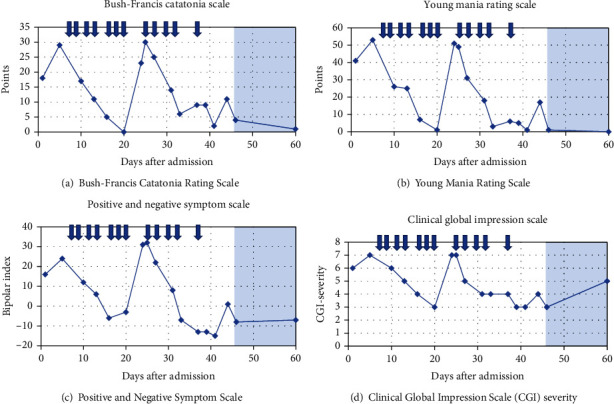
The figures illustrate the clinical rating of our patient's psychiatric status during her stay at the Child & Adolescent Psychiatry, Emergency Unit in Malmö. The ECT sessions are indicated with arrows and the colorized area represents time after discharge.

On admission to the emergency room, our patient was agitated, had disorganized, incomprehensible speech and incongruent symptoms with fluctuations between irrational laughing and crying. She had auditory hallucinations, a high pulse, and hyperventilation. She accepted 10 mg olanzapine and 20 mg alimemazine and was admitted to the emergency ward. During the first six days, our patient developed visual hallucinations, echolalia, and sexual, persecutory, and telepathic delusions, despite olanzapine (2 × 10 mg/day) and 20-40 mg alimemazine prescribed as needed. Her symptomatology, which combined manic and psychotic symptoms, was therefore considered coherent with an acute manic episode with psychotic features and fulfilled all criteria for a manic episode according to DSM-5 [6]. Oral treatment with antipsychotics and lithium was subsequently offered but refused. Further care was given according to the Swedish Compulsory Mental Care Act, and our patient was given intramuscular injections with 50-100 mg zuclopenthixol depot, 10-20 mg diazepam, and 5 mg biperiden on two occasions with a two-day interval. On the third day, she accepted lithium, but the adherence was low. Our patient responded to the treatment with greater tiredness and improved sleep but did not improve in terms of the manic symptoms. Instead, she developed signs of catatonia with disorganized motoric behavior including mannerisms and grimaces, staring, and agitation. Figures 1(a)–1(d) present the symptom progress during the treatment at the ward and at follow-up, including repeated assessments using the Bush-Francis Catatonia Rating Scale [16], Young Mania Rating Scale (YMRS) [17], Positive and Negative Symptom Scale (PANS-S) [18], and Clinical Global Impression–Severity Scale (CGI) [19]. Blood samples and a urine toxicology screen were taken, and a medical examination was performed. After initiation of compulsory injections, multidisciplinary clinical discussions regarding further treatment options were held with participating physicians from the departments of child and adolescent psychiatry, pediatric neurology, pediatric endocrinology, and adult psychiatry. A decision on ECT initiation was made five days after admission, after a dialogue with our patient and her parents. To exclude organic causes, a computed tomography (CT) scan, electroencephalography (EEG), analysis of cerebrospinal fluid (CSF), and electrocardiography (ECG) were performed in narcosis before treatment with ECT. A broader somatic investigation was also performed, including investigations for paraneoplastic syndrome and metabolic or autoimmune encephalitis. This included ultrasonography of the abdomen, X-ray of the thorax, gynecological examination, extended blood testing for inflammatory diseases, and screening for bacteria and virus in the urine and blood. The results showed an increased inflammatory response (erythrocyte sedimentation rate 30-57) and slightly increased levels of thyreoperoxidase (TPO) antibodies (110 kilo units/liter), as well as a vitamin D and iron deficiency. All other blood tests and examinations were normal, including tests for porphyria and for autoimmune encephalitis. Levels of thyroid-stimulating hormone (TSH) and thyroxine were also normal. Since our patient refused to take lithium, it was withdrawn, and olanzapine was switched to 20 mg aripiprazole daily to reduce risk of metabolic side effects of olanzapine, which is especially of importance in already obese patients [20].

Seven days after admission, bitemporal ECT was initiated under propofol sedation every 2-3 days, with a dosage of 205 to 230 millicoulombs (mC) and a pulse width of 0.4 milliseconds (ms). The average seizure length was 23.2 seconds. Our patient was clinically markedly improved after two ECT sessions (Figures 1(a)–1(d)). The treatment regime was therefore shifted to right unilateral ECT according to d'Elia [21] after five sessions, 18 days after admission. Side effects included headache and a slight temporary memory loss. There were no other adverse events. After seven ECT sessions, she was clearly improved (Figures 1(a)–1(d)), and it was decided to taper ECT sessions to every 4-5 days with termination after nine sessions. During this period, she was allowed to go home for half a day and spent time with her family.

Four days after the seventh ECT, day 24 after admission, our patient relapsed with manic and psychotic symptoms and agitation. She acted out on staff, had sexual persecutory delusions, and believed that some staff members were her late grandmother or other family members. She had disorganized motoric behavior, went back and forth in the corridors, reported auditory hallucinations, seemed afraid, and had disorganized speech and thoughts with echolalia and logorrhea. Somatically, she had an elevated body temperature, hyperventilation, and tachycardia with a heart rate of 150 beats/minute. She received an injection of 100 mg zuclopenthixol, 10 mg diazepam, and 5 mg biperiden. The day after, day 25, she received the eighth ECT with a slightly increased electrical stimulus (230.4 mC) and after two additional ECT treatments, along with increased aripiprazole dosage to 20 mg/day and lithium reintroduction, our patient's psychiatric status improved once again. The number of planned ECT treatments was extended to twelve. During the improvement after eleven ECTs, she had a more negative symptomatology with a negative bipolar index, as calculated by the PANS-S rating scale (Figure 1(c)). After finalization of the extended ECT regime, the manic state was broken, and she was discharged to outpatient care 46 days after admission.

### 2.3. Follow-Up Period

After discharge, our patient was closely monitored by colleagues in a bipolar and psychosis team, initially several times per week. On the family's request, lithium was withdrawn one year after discharge. Additional somatic and psychiatric investigations were conducted in the outpatient clinic. The erythrocyte sedimentation rate remained moderately elevated, but extended investigations for inflammatory diseases were negative, and it was therefore considered an unspecific marker of inflammation. It had been noted that our patient had a significant weight gain without a concomitant increase in length at age 12-14. Initially, due to an irregular menstrual cycle, menorrhagia, fatigue, and increased appetite, it was hypothesized that she might have a hormonal disorder. It was also noted that she had low-set ears and a short neck, and she had undergone reconstructive surgery for protruding ears as a child. The psychiatric evaluation revealed that she had developmental-related symptoms including social reservation, and a cognitive screening with Montreal Cognitive Assessment (MoCA) [22] revealed a low visuospatial ability. A genetic investigation was therefore performed. The results showed a partial loss in one copy of the X-chromosome, 46X, del (X)(p21), consistent with TS. Genetic analysis of the parents revealed that our patient's mother had a mosaicism for TS in peripheral blood cells (45X/46XX), but no definite genetic link between our patient's and her mother's karyotype was found. At the age of 18, she was discharged to the adult psychiatric clinic for further follow-up regarding bipolar disease and to the department of medicine for further somatic follow-up regarding TS. Since discharge, our patient has been admitted twice to the adult emergency psychiatry for suspected manic episodes. Apart from these episodes, she has been feeling well for several months. Three days a week, she works on completing her high school grades. Lithium has been reintroduced, with the same blood concentration as she had at discharge from the child and adolescent emergency unit.

## 3. Discussion and Conclusions

This is the first study to report that ECT could be effective in patients with TS and an acute manic episode and one of very few published reports on a girl with TS who develops an acute manic episode with psychotic features. TS is a rare condition that, due to its often nonspecific symptoms and lack of knowledge about its clinical presentation, often is undiagnosed in childhood and adolescence [1]. This case illustrates the importance of a comprehensive differential diagnostical reasoning and performing further somatic examinations when the clinical picture is atypical.

TS has been associated with a higher frequency of a range of psychiatric conditions such as anxiety, depression, and schizophrenia [7, 10, 12]. However, little research has focused on TS and bipolar disorders [10]. A previous case report on TS and schizoaffective syndrome was conducted on a patient with Turner mosaicism who, like our patient, was diagnosed in late adolescence after a period of grandiose delusions and bizarre behavior [8]. There are currently many theories regarding the association between psychotic disorders, affective disorders, and X-linked diseases, but without consensus [8, 10, 12]. Interestingly, a polymorphism in the *HOPA* (human opposite paired) gene, whose gene product serves as a cofactor for activation of the thyroid hormone receptors in Xq13, has been associated with psychosis and hypothyroidism, and the gene product has been associated with the pathogenesis of TS [23]. TS has also been linked to differences in brain volume, which could explain parts of the psychological functional profile in affected individuals. In a study by Murphy et al., 18 TS patients were investigated with magnetic resonance imaging (MRI) and the volume of the basal ganglia, parietooccipital lobe, and hippocampus was found to be decreased compared to controls, suggesting that the X-chromosome is significantly linked to brain development [24]. These findings offer a plausible anatomical explanation for the visuospatial impairment seen in TS patients [25]. Hart et al. performed a case-control study and showed that TS patients have a decreased recruitment of frontoparietal circuitry during visuospatial processing, visualized with functional MRI, further indicating an importance of the X-chromosomes in brain function [26]. However, the decreased volume in brain structures and difference in function described in the studies may be a result of a combination of genes and endocrine factors, and the impact of decreased brain volume on the neuropsychiatry of TS patients still needs more research [24].

There is growing evidence that autoimmune processes could play a causative role in several neuropsychiatric disorders, including psychosis [27] and possibly bipolar disorder [28]. Girls with TS are also at increased risk of developing a wide range of autoimmune diseases, especially thyroid diseases, but also celiac disease, type 1 diabetes mellitus, rheumatoid diseases, and inflammatory bowel disease [1, 29]. The exact etiology behind the increased risk of autoimmunity remains unsolved. In a Danish study, several kinds of autoantibodies (against gliadin, transglutaminase, adrenal cortex, intrinsic factor, TPO, and glutamic acid decarboxylase) were found in most women with TS [30]. TPO antibodies have been associated with encephalopathy, then often referred to as Hashimoto's encephalopathy (HE). However, antithyroid antibodies can also be found in healthy children and are at present not considered pathogenic in HE; they are instead regarded as markers of a general susceptibility to autoimmunity [31, 32]. Our patient had an elevated TPO antibody titer with normal thyroxine values, absence of autoantibodies or pleocytosis in the cerebrospinal fluid (CSF), normal EEG findings, and normal CT scan of the brain. The finding of TPO antibodies was in our patient considered nonspecific, but the relation between autoimmunity and bipolar disease, psychosis, and encephalopathy is not yet fully understood.

Before antimanic treatment is initiated, a differential diagnostic reasoning is essential, and somatic comorbidity such as anti-NMDAR encephalitis or HE must be ruled out. A subcommittee of the Autoimmune Encephalitis International Working Group has recently proposed an algorithm and redefined the criteria for autoimmune encephalitis in children [31]. The clinical picture, further diagnostics, and treatment procedure should ideally be discussed by an interdisciplinary team. Genetic investigations may also be of importance to fully understand the clinical picture, with the goal of personalized medicine and avoiding unnecessary side effects.

When treating severe mania, guidelines suggest a trial of two consecutive antipsychotics at the maximum licensed dose, followed by adding lithium if needed [33]. ECT is recommended when other treatment options have proven ineffective, or in potentially life-threatening conditions, based on an individual risk-benefit assessment. The symptoms of mania in adolescents may profoundly impact on their life, and there is often a need to resolve the manic episode quickly. In addition, children and young people in general are more sensitive than adults to the potential adverse effects of antipsychotics. Some previous case reports on women with TS and a psychiatric syndrome have described both diagnostic and treatment difficulties [10]. As ECT is recognized as being efficient and safe both in adults and in adolescents when the indication is correct [13, 14], we suggest it should be considered more often and earlier in children and adolescents, also with genetic syndromes. A rapid relief from a manic state is considered beneficial for the brain [14], and ECT has the potential to provide a more rapid response with fewer side effects than psychopharmacological agents [15]. In patients with TS, drugs with hormonal and metabolic side effects such as hypothyroidism or weight gain should especially be avoided. Our patient responded rapidly to the given treatment, with transient short-term memory loss and headache as the only adverse events. When the time between the ECT sessions in the index series was prolonged, she relapsed. Thus, to taper out ECT too prematurely might jeopardize the treatment result and a gradual tapering until remission depending on the patient's symptoms could prevent relapses in children, as shown earlier in adults with depression [34]. A strength of this report is the repeated assessments of our patient's symptoms, systematically registered with a good temporal resolution during her time at the ward, with a two-year follow-up period.

## 4. Conclusions

The present case illustrates that ECT may be a safe and efficient treatment strategy for acute manic episodes in children and adolescents with concomitant TS and that severely affected adolescents may require a prolonged index series with gradual tapering of ECT. The present case also demonstrates a possible association between TS and bipolar syndrome. Finally, this case reveals important diagnostic and treatment challenges where the clinical presentation of a manic episode in a patient with this TS could be more complex and the treatment response slower.

## Data Availability

The data used to support the findings of this study are included within the paper.

## Abbreviations

ADHD: Attention deficit hyperactivity disorder
CGI: Clinical Global Impression
CSF: Cerebrospinal fluid
CT: Computed tomography
DSM: Diagnostic and Statistical Manual of Mental Disorders
ECG: Electrocardiography
ECT: Electroconvulsive therapy
EEG: Electroencephalography
HE: Hashimoto's encephalopathy
HOPA: Human opposite paired
mC: Millicoulombs
mg: Milligram
MoCA: Montreal Cognitive Assessment
MRI: Magnetic resonance imaging
Anti-NMDAR: Anti-N-methyl-D-aspartate receptor
PANS-S: Positive and Negative Symptom Scale
TPO: Thyreoperoxidase
TS: Turner syndrome
TSH: Thyroid-stimulating hormone
YMRS: Young Mania Rating Scale.
